# Assembly of a gene sequence tag microarray by reversible biotin-streptavidin capture for transcript analysis of *Arabidopsis thaliana*

**DOI:** 10.1186/1472-6750-5-5

**Published:** 2005-02-03

**Authors:** Valtteri Wirta, Anders Holmberg, Morten Lukacs, Peter Nilsson, Pierre Hilson, Mathias Uhlén, Rishikesh P Bhalerao, Joakim Lundeberg

**Affiliations:** 1 Department of Molecular Biotechnology, KTH-Royal Institute of Technology, AlbaNova University Center, SE-106 91, Stockholm, Sweden; 2 Department of Plant Systems Biology, VIB – Ghent University, B-9052 Ghent, Belgium; 3 Department of Forest Genetics and Plant Physiology, The Swedish University of Agricultural Sciences, SE-901 83, Umeå, Sweden

## Abstract

**Background:**

Transcriptional profiling using microarrays has developed into a key molecular tool for the elucidation of gene function and gene regulation. Microarray platforms based on either oligonucleotides or purified amplification products have been utilised in parallel to produce large amounts of data. Irrespective of platform examined, the availability of genome sequence or a large number of representative expressed sequence tags (ESTs) is, however, a pre-requisite for the design and selection of specific and high-quality microarray probes. This is of great importance for organisms, such as *Arabidopsis thaliana*, with a high number of duplicated genes, as cross-hybridisation signals between evolutionary related genes cannot be distinguished from true signals unless the probes are carefully designed to be specific.

**Results:**

We present an alternative solid-phase purification strategy suitable for efficient preparation of short, biotinylated and highly specific probes suitable for large-scale expression profiling. Twenty-one thousand *Arabidopsis thaliana *gene sequence tags were amplified and subsequently purified using the described technology. The use of the arrays is exemplified by analysis of gene expression changes caused by a four-hour indole-3-acetic (auxin) treatment. A total of 270 genes were identified as differentially expressed (120 up-regulated and 150 down-regulated), including several previously known auxin-affected genes, but also several previously uncharacterised genes.

**Conclusions:**

The described solid-phase procedure can be used to prepare gene sequence tag microarrays based on short and specific amplified probes, facilitating the analysis of more than 21 000 *Arabidopsis *transcripts.

## Background

Extensive transcriptional profiling of the plant model system *Arabidopsis thaliana *has been limited when compared to other model organisms, such as human and mouse, mainly due to the lack of high-quality cDNA microarrays offering genome-wide coverage. However, during the recent years both academic and commercial alternatives to these cDNA arrays have emerged. The public initiative by the CATMA consortium has aimed at the production of high-quality probes for each of the 29 787 genes predicted in the *Arabidopsis *genome [1,2]. The design of the CATMA gene sequence tag (GST) probes is based on de novo gene prediction from the genome sequence [1,3,4], since only a relatively now number of ESTs are available for Arabidopsis (dbEST at NCBI contains only about 320 000 *Arabidopsis *ESTs compared with 6 million for the human species). Commercial alternatives for genome-wide monitoring of the *Arabidopsis *transcriptome have been developed by Affymetrix, Agilent Technologies, MWG Biotech, Operon and others. In a recent study the CATMA, Affymetrix and Agilent arrays were found to perform equally, but with a minor advantage for the CATMA arrays in terms of dynamic range [5].

In the first phase of the CATMA program 21 120 GSTs covering more than 70% of the predicted genes were designed. The length of the probes is kept low to ensure specificity and ranges from 150 bp to 500 bp, with the size distribution heavily shifted towards the shorter fragments. To further increase the specificity of the GSTs their distribution is shifted towards the 3'-end of the genes, with 60%, 16% and 24% representing 3'-, centre and 5'-regions, respectively [1,3]. Both 3' and 5' untranslated regions of the genes were included in the design. As a consequence of the GST fragment length, an efficient and robust high-throughput method for purification of short fragments is needed. Here we demonstrate that the purification can be accomplished by taking advantage of the recent finding that the streptavidin-biotin bond can be broken, in a fully reversible fashion, without denaturation of the protein [6]. The approach is based on incorporation of a biotin molecule during PCR amplification of the GSTs, binding of the products to streptavidin-coated paramagnetic beads using high ionic-strength conditions and elution through disruption of the streptavidin-biotin bond in a non-denaturing and fully reversible fashion with deionised water. We exemplify that this feature can be applied for generation of high-quality gene sequence tag microarrays in a cost-effective and high-throughput manner. We also demonstrate the use of these arrays by presenting results on the alteration in gene expression levels at different time points in *Arabidopsis *plants treated with physiological concentrations of the well-known plant hormone indole-3-acetic acid (auxin). Finally, we compare our results with those obtained in two previous studies [7,8] carried out on the Affymetrix 8 k Gene Chip platform to identify auxin regulated gene expression.

## Results and discussion

In this study we present a method suitable for purification of gene sequence tags, which have recently been designed and successfully used for transcriptional profiling of the plant model system *Arabidopsis thaliana *[1,3]. The purification method is based on reversible biotin-streptavidin binding, utilises streptavidin-coated paramagnetic beads and can be automated on a robotic workstation dedicated for magnetic separation and equipped with a temperature control [6]. We exemplify the performance of the method by studying the purification of three representative biotinylated amplification products and subsequently show that arrays prepared using this method can successfully be used for large-scale transcriptional profiling.

**Figure 1:**
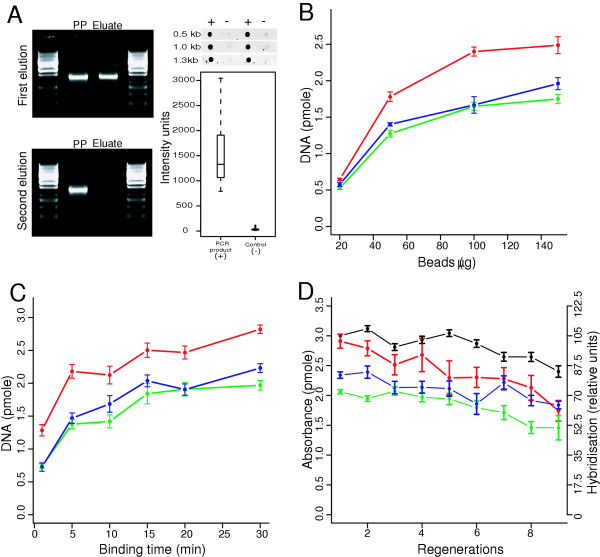
Solid-phase purification parameters. In **A **the agarose gel image shows the first (upper left image) and second (bottom left image) elution of the captured 1.0 kb product. The first and fourth lanes contain a size marker, while the second and third lanes contain an equal amount, assuming 100% yield at purification step, of unpurified PCR product (PP) and purified PCR product (eluate), respectively. The images on the right shows the results from the carry-over test, where PCR product and water were used as input samples for multiple consecutive purification reactions in an alternating order. The upper part shows the hybridisation results (PCR product, water, PCR product and water), while the box-and-whiskers plot below shows the quantifications of the signals (n = 18, six replicates of the three different products). Purification of three amplification products (red line 0.5 kb, green line 1.0 kb and blue line 1.3 kb) is investigated using an increasing amount of streptavidin-coated magnetic beads (number of independent replications, n = 8, panel **B**), varying binding time (n = 4, panel **C**) and repeatedly used beads (n = 4, panel **D**). The black line in (**D**) is based on fluorescence data and is plotted using the y-axis on the right, while the three other lines are based on absorbance measurements and use the y-axis on the left side. The presented data originates from repeated independent experiments and the error bars denote the calculated standard error.

The amplification products we use to study the purification process are 500 bp, 1 kb and 1.3 kb in length, covering the size range typically used for probes on cDNA arrays. For successful purification of the probe both efficient capture by the beads and release is important. An example of the capture of a 50-μl PCR product and subsequent release is shown in Figure 1A. As shown, the initial capture and release is highly efficient (upper left panel), and with no product detectable in the eluate corresponding to the second release (lower left panel). Next we analyse the efficiency of the capture reaction using an increasing amount of beads while keeping the amount of PCR product and length of incubation time constant. The results indicate that for a standard 50-μl PCR product highly efficient capture is achieved already at approximately 100 μg of beads for all products up to 1.3 kb (Figure 1B), but for a highly optimised amplification reaction a higher amount of beads may be necessary (data not shown). As expected, the binding of the biotinylated product to the streptavidin moiety is a rapid and efficient process with the majority of the binding taking place during the first minutes of incubation (Figure 1C). Shorter products appear to have faster binding kinetics reaching saturation at earlier time points. Also important to note is that the molar amount of captured and eluted product is not equal for the different-sized products, indicating that other factors such as steric hindrance also contribute to capacity of the beads and should be considered for purification of longer products > 1000 bp.

Repeated use of the magnetic beads after a single round of capture and elution is the key feature of the described strategy. To investigate the cross-contamination between iterative cycles of purification as well as the total number of bead purifications that can be used without significant loss in performance, we used agarose gel electrophoresis, DNA Lab-on-chip technology as well as a more sensitive approach based on printing of eluates onto glass slides and hybridisation using a fluorescently labelled oligonucleotide complementary to the purified and printed probes. A carry-over free purification requires that all captured product is released at the first elution so that no product is transferred to the next sample to be purified using the same set of beads. We analysed the presence of cross-contamination by analysing the eluates of two consecutive release reactions from a single immobilised product (Figure A, left panels). Furthermore, the cross-contamination issue was analysed by using the reversible beads in six sequential capture reactions containing either a PCR product or a water-only control in an alternating order (Figure A, right panel). A released product is detected in the first eluate, as expected, but not in the second, by all three methods including the sensitive fluorescence assay. As shown in Figure 1A, right panel, hybridisation with a labelled oligonucleotide complementary to the purified probes shows a signal in features originating from a PCR product of the three sizes, but not in features originating from the negative water-only control.

We studied the capacity of the beads after multiple capture and release cycles by using a constant amount of the three PCR products as input for each iterative purification cycle. An extra washing step was carried out between the purification cycles. Data for nine consecutive binding, washing, elution and regeneration rounds of the PCR products is presented in Figure 1D. The yields of purified products are similar for six rounds of reuse with a minor decline during the subsequent cycles, which more likely correlates with loss of beads during the washing steps than with reduced capacity. We continued to analyse the efficiency of the bead regeneration and reuse by using amplification products of twelve additional clones (range 0.3 – 2 kb) and a hybridisation-based quantification approach. The clones were amplified, purified using beads reused up to nine times, printed onto glass slides and finally hybridised with a DNA-binding dye to determine the amount of purified product. A clone-wise scaling of the hybridisation signal of each subsequent reuse versus the signal corresponding to the first use was carried out, followed by a calculation of the overall average, which is shown as the solid black line in Figure 1D. The results from the quantification through hybridisation are in close agreement with the pattern observed using the probes discussed in more detail above.

### Assembly of the Arabidopsis gene sequence tag microarray

We applied the described method for purification of 21 120 *Arabidopsis *biotinylated gene sequence tags (GSTs), with sizes ranging from 150 to 500 bp. The use of GSTs in transcript profiling offers improved specificity when compared to the more common EST or cDNA libraries since each GST has been designed to have minimal cross-hybridisation to other genes, including members of the same gene family. The investigated set of GSTs covers approximately 70% of the genes in the genome, as described in more detail by the CATMA consortium [1]. The consortia amplification strategy is based on a two-step PCR system. This facilitates, as shown in this study, the incorporation of a biotin label in the second PCR by generic handle sequences introduced at the initial amplification step. This circumvents the need to design individual gene-specific biotinylated primers. The products were purified in an automated fashion onto 200 micrograms of magnetic, streptavidin-coated beads that were reused up to six times. To compensate for the higher molar amount of the GST amplification products, an initially higher amount of beads was used for the purification of the GSTs than was used for the optimisation of the method. After elution with 12 μl water an equal amount of DMSO is added to eluted products, which are then printed onto the glass slides.

### Changes in gene expression caused by auxin treatment

**Figure 2:**
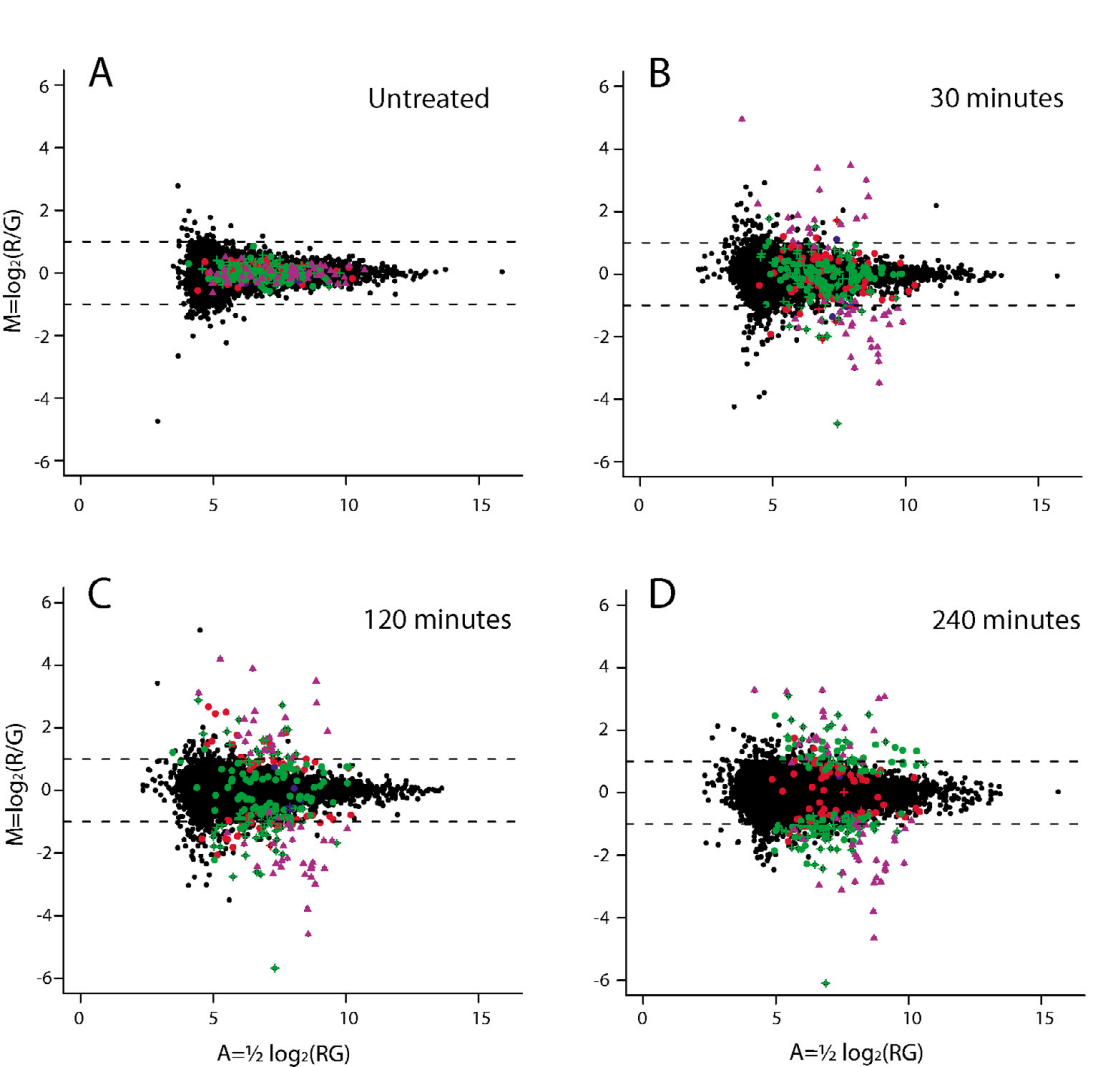
General overview of the filtered and normalised data for each of the time point comparisons. (**A-D**) The average of the replicated hybridisations for each time point is presented using MA-plots (samples are labelled with a **R**ed dye and the reference with a **G**reen dye). An increased noise at lower signal intensities is observed, as expected, as no absolute cut-off level for signal intensity is used in the data filtering process. However, genes at the low-intensity region are not identified as differentially expressed genes due to variation between the replicated hybridisations. (**B**) At 30 minutes the overall pattern is drastically changed, with several genes showing a >2-fold change in expression levels. (**C-D**) The same general pattern is observed for the data corresponding to 120- and 240-min treatments. An increasing number of differentially expressed genes are, however, observed. For (**A-D) **genes differentially expressed at one time point ● (30 min blue, 120 min red, 240 min green), two time points + (30 and 120 min red, 30 and 240 min blue, 120 and 240 min green) or three time points ▲ (purple) time points are listed in Additional data files 1 and 2 available online. Dashed lines indicate two-fold differential expression.

The arrays generated by large-scale purification of GSTs are used in a pilot time-point study where the plant hormone indole-3-acetic acid (IAA), also known as auxin, is used to cause transcriptional changes in *Arabidopsis thaliana *seedlings. Total RNA is collected at three post-treatment time points and compared, using a reference design, to RNA extracted from untreated plants. A general overview of the data is shown in Figure 2. Using the filtered and normalised data (for details see Materials) genes which are differentially expressed upon auxin treatment are identified using a Bayesian approach [13,14]. Using a false discovery rate adjusted p-value of less than 0.001 as threshold level for differential expression, a total of 120 and 150 genes are found to be up- and down-regulated, respectively, at one or more of the three time points (see Additional data files 1 and 2). As expected, none of these genes are differentially regulated in the control self-to-self hybridisation experiment (Figure 2A). It is previously known that auxin influences several key processes during plant growth and development and several lines of evidence indicate that auxin regulation of gene expression plays a key role in its mode of action. A particularly well-studied pathway of auxin-regulated gene expression is the auxin induction of the *Aux/IAA *genes. The *Aux/IAA *genes encode small short-lived nuclear proteins that interact with the ARF (auxin response factor) family of transcription factors and are thought to modulate the transcriptional activity of the ARFs in an auxin-dependent manner. These ARFs have been shown to bind to auxin-responsive elements (AuxREs) that are found in promotors of several auxin-regulated genes [16]. The ARFs function as both transcriptional activators and repressors [17], and the combination of ARF and Aux/IAA proteins is thought to mediate the tissue-specific effects of auxin [18,19]. Thirteen of the up-regulated genes identified in this study are previously shown to be auxin-regulated and include several members of the Aux/IAA family. These exhibit different induction patterns, with for example *IAA5 *and *IAA19 *being strongly up-regulated (>30- and 10-fold, respectively) already at 30 minutes, while a two-hour treatment is required for the *IAA7 *transcripts to reach a two-fold up-regulation. In a recent independent study where *Arabidopsis *seedlings were treated for only 15 min with 1 μM IAA, all of the *Aux/IAA *genes listed in Additional data file 1, with the exception of *IAA7*, were found to be up-regulated [7]. In addition to the *Aux/IAA *genes four members of the GH3 family that have also been shown to be induced by auxin exhibited a rapid and sustained 2- to 8-fold up-regulation in our study, again confirming the findings reported in a previous study on auxin regulation of gene expression using Affymetrix 8 k oligonucleotide arrays [8]. A key feature of auxin regulation of development is the polar transport of auxin that is mediated by auxin transporters. Our data indicate that the polar auxin transporters *PIN1 *[20] and *PIN7 *are up-regulated by auxin whereas in contrast the expression of several members of the Aquaporin gene family [21] are down-regulated. Expression of the PIN transporters is up-regulated already at 30 minutes and remains high throughout the studied time frame. These observations, of control of auxin transporters by auxin, are interesting since it is known that auxin regulates its own transport but to date there has been little data on this type of feedback. Other genes that are influenced by auxin in our study include transcription factors (8 up-regulated and 15 down-regulated), genes involved in signal transduction (7 and 6, respectively), metabolic enzymes (15 and 30, respectively), as well as several genes classified to other categories and also currently unknown genes. The most down-regulated gene at all time points (CATMA5a08790), for example, shows no sequence similarity to any known sequence and has no recorded expression in any of the sequenced tissue libraries deposited into the public domain. These 270 genes are interesting candidates for further research, but it is important that additional validations are carried out to identify and separate the immediate auxin target genes from the indirect.

## Conclusions

We have described an efficient procedure for large-scale purification of gene sequence tags that can be used for several purposes including microarray fabrication. We demonstrate the utility of the technology by applying it to generate more than 21 000 short (150 – 500 bp) and highly specific *Arabidopsis *gene sequence tags for use as microarray probes in transcriptional profiling. Biotinylated amplification products are rapidly captured and eluted using a reusable streptavidin-coated solid-phase support in an automated high-throughput manner directly compatible with subsequent microarray printing. Our results demonstrate that the assembly and purification of gene-specific tags is an alternative to currently used purification methods, especially suitable for short amplification products such as gene sequence tags. In addition, the possibility to generate single-strand probes in the range of 150–500 nucleotides by a sodium hydroxide treatment of immobilised probes with subsequent elution of the remaining biotinylated strand, opens up for new microarray applications that would extend probe length beyond current oligonucleotide synthesis limits.

## Methods

### Optimisation of the purification procedure

The performance of the described purification method was investigated by varying either the amount of beads, the length of the incubation time for binding of the biotinylated product to the streptavidin-coated beads and the number of times the beads were reused. We also investigated if multiple reuses of the beads did introduce a well-to-well cross-contamination. The section below describes the general aspects of the purification and is followed by a more detailed description of the experiments carried out to investigate the different above-mentioned aspects of the purification method.

For all experiments three randomly chosen EST clones (0.5 kb, 1 kb and 1.3 kb) were amplified in 50-μl reactions containing 20 mM Tris-HCl, pH 8.4, 50 mM KCl, 1.5 mM MgCl_2_, 200 nM dNTPs (Amersham Biosciences Europe GmbH, Sweden), 5 pmole universal sequencing primer (USP, 5'-TAAGCTAGGCACTGGCCGTCGTTTTACAACG-3', MWG Biotech AG, Germany), 5 pmole biotinylated reverse sequencing primer (RSP, 5'-AGGCCTAATGGTCATAGCTGTTTCCTGTGTG-3', MWG Biotech AG) and 1.5 units Platinum *Taq *DNA Polymerase (Invitrogen AB, Sweden). The temperature cycling (5 min at 95°C, 30 × (30 s at 95°C, 30 s at 64°C, 2 min at 72°C), 10 min at 72°C) was carried out in a Hybaid thermal cycler (Thermo Electron Molecular Biology, MA, USA). Pooling and splitting into aliquots of 50 μl in a clone-wise manner was used to remove variances introduced by the amplification step.

All purification steps, including bead dispensing, binding of the biotinylated product to the streptavidin moieties, washing, elution and regeneration of beads, were carried out in the Magnatrix 1200 automated workstation (Magnetic Biosolutions AB, Sweden). The biotinylated amplification products were bound to Dynabeads M-270 Streptavidin beads (Dynal Biotech ASA, Norway) during an incubation at room temperature using a high-salt binding buffer [1 M NaCl, 10 mM Tris-HCl, pH 7.5, 1 mM EDTA, 5% PEG-6000 and 0.1% Tween-20] and when bound, washed with 1 × TE-buffer [10 mM Tris-HCl, pH 7.5, 1 mM EDTA]. During incubation the beads were kept in suspension by mixing through pipetting every third minute. Elution was achieved by breaking the streptavidin-biotin bond in a 20-μl volume using deionised H_2_O. By use of a peltier thermal element, the immobilised products kept in suspension were heated in deionised water to 80°C (1°C / 2 s) for 1 second and cooled to room temperature (1°C / 2 s). Efficient elution is achieved through a combination of elevated temperature, appropriate temperature ramping and incubation at the elevated temperature, as described in more detail elsewhere [6]. The beads were separated from released products by magnetic separation, reconditioned through a repeated wash procedure with 1 × TE-buffer and, finally, prepared for the next round of purification. Quantification of DNA was carried out using the Nanodrop ND-1000 spectrophotometer (NanoDrop Technologies Inc, DE, USA).

For all the purifications described below an aliquot of the pooled PCR product corresponding to a 50-μl reaction was used. The binding capacity of the beads was studied using an increasing amount (10 μg, 50 μg, 100 μg and 150 μg) of fresh beads (first use) while keeping the incubation time constant at 20 minutes. To estimate the variability of the method eight independent purifications were carried out (n = 8). The effect of the length of the incubation time was studied using 150 μg of beads and eight different incubation times (1, 5, 10, 15, 20 or 30 min) with four replicates of each (n = 4). To study the effects of multiple reuses of the beads, the same beads were reconditioned and used up to nine times. The amount of beads in the first capture was 150 μg and the capture time 20 minutes. The variability was estimated using four independent replications. The multiple reuse of the beads was also analysed using a hybridisation based approach. Twelve clones ranging from 0.3 to 2 kb were amplified and purified multiple times using reconditioned beads. The purified products were subsequently printed in eight replicates onto glass slides and quantified using Syto61 (Molecular Probes Inc, OR, USA). The well-to-well carry-over of product was analysed by first purifying one of the PCR products, followed by a purification reaction with no PCR product added (water-only control). This pattern was repeated six times for all three products, while the same set of beads was used for all purifications. Eluates from all these purifications were printed on slides and hybridised with a Cy5-labelled oligonucleotide complementary to the common vector sequence present in all products. Hybridisation was carried out using 10 pmole of the labelled oligonucleotide for 1 hour at 35°C in a hybridisation solution containing 50% formamide, 5 × SSC and 0.1% SDS. Slides were washed with 2 × SSC containing 0.1% SDS (5 min at room temperature) and three times with 1 × SSC (1 min at room temperature). Scanning using the G2565BA DNA microarray scanner (Agilent Technologies) was carried out at the highest possible photo multiplier tube setting in order to reveal low-level signals.

### Preparation and arraying of gene sequence tags

Initial amplification from BAC-clones or genomic DNA was carried out by the CATMA consortium at different nodes throughout Europe [1,2]. One percentage of the first amplification product, obtained using gene-specific primers with 5' handle sequences, was used as template for the second amplification. A total of 51 cycles [11 × (15 s at 94°C, 15 s at 55°C (-1°C / cycle), 30 s at 72°C), 40 × (15 s at 94°C, 30 s at 55°C, 30 s at 72°C)] were carried out in the presence of 20 mM Tris-HCl, pH 8.4, 50 mM KCl, 1.5 mM MgCl_2_, 200 nM dNTPs, 20 pmole of each primer (forward primer biotinylated, Thermo Hybaid GmbH, Germany) and 1.5 units Platinum *Taq *DNA Polymerase in a total volume of 50 μl. Biotinylated products from each amplification reaction were bound to 200 μg of Dynabeads M-270 Streptavidin beads (reused up to six times) during a 15-minute incubation at room temperature using a high ionic-strength buffer, washed with 1 × TE-buffer and, finally, eluted with 12 μl deionised water. After each capture round the beads were reconstituted, pooled plate-wise and randomly assigned to a new plate. An equal volume of 99.9% dimethyl sulfoxide (DMSO) (Sigma-Aldrich Sweden AB, Sweden) was added and the purified amplification products were arrayed into 22 by 22 patterns in 48 individual blocks with the QArray arrayer (Genetix Limited, UK) and SMP2.5 pins (TeleChem International Inc, CA, USA). When dried, printed products were immobilised to the reactive surface of the Ultra-GAPS slides (Corning B.V. Life Sciences, The Netherlands) using 250 mJ/cm^2 ^UV-light (Stratalinker, Stratagene Europe, The Netherlands).

### Auxin treatment and sample preparation

10-day-old *Arabidopsis *Col-0 seedlings were grown at 22°C in MS medium (Duchefa AB, The Netherlands) supplemented with 0.5% sucrose and using a 24-h photoperiod with 16 h of light at 75 mE m^-2 ^sec^-1 ^PAR. The samples were treated with 1 μM indole-acetic acid for a period of 0, 30, 120 and 240 minutes, washed once with an excess of MS medium with 0.5% sucrose for 5 minutes, frozen in liquid nitrogen and stored at -70°C. For each time point, frozen seedlings from three independent vials were pooled, grinded and total RNA extracted using the RNeasy kit (Qiagen GmbH, Germany). The quality of the RNA was determined using the RNA 6000 Nano kit and the Bioanalyzer instrument (Agilent Technologies, CA, USA).

### Target labelling, hybridisation, washing and scanning

Ten μg anchored oligo dT primer (dT_20_VN, MWG Biotech AG) was annealed to 20 μg total RNA after a denaturating step (10 min at 70°C). The cDNA synthesis reaction was carried out at 42°C for 1 h 45 min in a 30-μl reaction containing 2 mM dNTPs (dTTP:aminoallyl-dUTP in 1:4, unmodified Amersham Biosciences, modified Sigma-Aldrich), first-strand buffer (50 mM Tris-HCl, pH 8.3, 75 mM KCl, 3 mM MgCl_2_), 0.01 mM DTT and 400 units Superscript II (Invitrogen AB). The synthesis reaction was terminated by addition of EDTA, the RNA strand hydrolysed with NaOH (15 minutes at 70°C) and the reaction neutralised with HCl (final concentrations 20 mM, 150 mM and 150 mM, respectively). The cDNA strands were purified using the MinElute spin columns (Qiagen GmbH) with the provided wash and elution buffers replaced by 80% ethanol and 100 mM NaHCO_3_, pH 9.0, respectively. Monofunctional NHS-ester Cy3 or Cy5 fluorophores (Amersham Biosciences) were coupled to the amino-allyl groups during a 90-minute incubation at room temperature after which unincorporated ester groups were inactivated through a hydroxylamine treatment (final concentration of 730 mM). The pooled labelling reactions were purified using MinElute spin columns and hybridised to the arrays using a two-step protocol in the GeneTac hybridisation station (Genomic solutions Ltd, UK). The pre-hybridisation at 42°C for 45 min (5 × SSC, 1% BSA (Sigma-Aldrich), 0.1% SDS, 40 μg poly(dA) (Sigma-Aldrich) and 20 μg tRNA (Sigma-Aldrich)) was followed by a 16–18 h hybridisation at 42°C with the labelled material and a hybridisation buffer containing 5 × SSC, 25% formamide, 0.1% SDS, 40 μg poly(dA) and 20 μg tRNA. The slides were washed with 2 × SSC and 0.1% SDS at 42°C, followed by 0.1 × SSC + 0.1% SDS at room temperature and finally by three repeated washes with 0.1 × SSC at room temperature. Slides were scanned at 10-μm resolution using the G2565BA DNA microarray scanner for which the photo multiplier tube (pmt) setting was adjusted so that the images for the Cy3 and Cy5 channels were in balance as determined by visual observation. Each time point-to-reference sample comparison was carried out on two arrays, with the dye labels exchanged between the replicated hybridisations in order to avoid sequence-dependent labelling and hybridisation effects. A control self-to-self hybridisation was also carried out for the untreated sample in order to assess the level of noise in the experimental system.

### Image processing and data analysis

The acquired tiff-images were processed using the GenePix 4.1 software (Axon instruments Inc, CA, USA) and the data with the R environment for statistical computing [9], Bioconductor [10] and the aroma package for microarray data analysis [11]. Expression values for each feature and dye channel were obtained by subtracting the median of the local background value from the median of the foreground value. Features for which the background subtracted value were zero or below in one of the channels, but not in the other, were given the expression value of 1. A feature was considered uncertain and removed from subsequent data analysis by setting its value to NA (not available) if a) it was flagged as Not Found by GenePix, b) it was manually flagged as bad (dust particles etc), c) the signals for both channels were saturated, d) the percentage of foreground pixels above the median background + 2 SD were below 60 for both channels or e) the feature diameter was <70 μm or >120 μm. Filtered data was normalised separately for each individual block on the slide using the intensity-dependent lowess method [12] and no between-slides scaling of the ratio values was deemed necessary. Differentially expressed genes were identified using a moderated t-test based on gene-wise standard errors estimated by an empirical Bayes method [13,14]. Genes with a false discovery rate adjusted p-value of less than 0.001 for any of the three time points were considered as potentially differentially expressed and are included in the Additional data files 1 and 2. The MIAME compatible data set, including processed and unprocessed data, is made available to the research community through the ArrayExpress expression data repository at the EMBL using the accession number E-MEXP-140 [15].

## List of abbreviations

CATMA a complete Arabidopsis thaliana transcriptome microarray

EST expressed sequence tag

GST gene sequence tag

IAA indole-3-acetic acid

MIAME minimum information about a microarray experiment

## Authors' contributions

VW carried out the laboratory work and the data analysis and participated in the design of the study and drafting of the manuscript. AH and ML participated in the solid-phase purification procedure. PN participated in the array production. PH designed and provided the CATMA probes. MU participated in the automation of the solid-phase procedure. RB carried out the auxin treatment and participated in the interpretation of the expression data. JL conceived of the study, participated in the drafting of the manuscript and coordinated the study. All authors read and approved the final manuscript.

## Supplementary Material

Additional File 1 List of genes identified as up-regulated by the indole-3-acetic acid treatment. Group labels refer to (A) previously identified auxin regulated, (B) transcription related, (C) signal transduction related, (D) transport, (E) cell wall establishment related, (F) metabolic enyzmes, (G) light related, (H) disease repsonse related, (I) other and (J) unknown genes. Click here for file

Additional File 2 List of genes identified as down-regulated by the indole-3-acetic acid treatment. Group labels refer to (A) previously identified auxin regulated, (B) transcription related, (C) signal transduction related, (D) transport, (E) cell wall establishment related, (F) metabolic enyzmes, (G) light related, (H) disease response related, (I) other and (J) unknown genes. Click here for file
